# Suppressive effect of epigallocatechin‐3‐O‐gallate on endoglin molecular regulation in myocardial fibrosis *in vitro* and *in vivo*

**DOI:** 10.1111/jcmm.12895

**Published:** 2016-06-16

**Authors:** Chiu‐Mei Lin, Hang Chang, Bao‐Wei Wang, Kou‐Gi Shyu

**Affiliations:** ^1^ Department of Emergency MedicineShin Kong Wu Ho‐Su Memorial HospitalTaipeiTaiwan; ^2^ Faculty of MedicineSchool of MedicineFu Jen Catholic UniversityTaipeiTaiwan; ^3^ Institute of Injury Prevention and ControlCollege of Public HealthTaipei Medical UniversityTaipeiTaiwan; ^4^ Central LaboratoryShin Kong Wu Ho‐Su Memorial HospitalTaipeiTaiwan; ^5^ Division of Cardiovascular diseasesDepartment of Internal MedicineShin Kong Wu Ho‐Su Memorial HospitalTaipeiTaiwan; ^6^ Institute of Clinical Medical SciencesCollege of MedicineTaipei Medical UniversityTaipeiTaiwan

**Keywords:** EGCG, angiotensin II, endoglin, JNK, AP‐1, myocardial fibrosis

## Abstract

Epigallocatechin‐3‐O‐gallate (EGCG), derived from green tea, has been studied extensively because of its diverse physiological and pharmacological properties. This study evaluates the protective effect of EGCG on angiotensin II (Ang II)‐induced endoglin expression *in vitro* and *in vivo*. Cardiac fibroblasts (CFs) from the thoracic aorta of adult Wistar rats were cultured and induced with Ang II. Western blotting, Northern blotting, real‐time PCR and promoter activity assay were performed. Ang II increased endoglin expression significantly as compared with control cells. The specific extracellular signal‐regulated kinase inhibitor SP600125 (JNK inhibitor), EGCG (100 μM) and c‐Jun N‐terminal kinase (JNK) siRNA attenuated endoglin proteins following Ang II induction. In addition, pre‐treated Ang II‐induced endoglin with EGCG diminished the binding activity of AP‐1 by electrophoretic mobility shift assay. Moreover, the luciferase assay results revealed that EGCG suppressed the endoglin promoter activity in Ang II‐induced CFs by AP‐1 binding. Finally, EGCG and the JNK inhibitor (SP600125) were found to have attenuated endoglin expression significantly in Ang II‐induced CFs, as determined through confocal microscopy. Following *in vivo* acute myocardial infarction (AMI)‐related myocardial fibrosis study, as well as immunohistochemical and confocal analyses, after treatment with endoglin siRNA and EGCG (50 mg/kg), the area of myocardial fibrosis reduced by 53.4% and 64.5% and attenuated the left ventricular end‐diastolic and systolic dimensions, and friction shortening in hemodynamic monitor. In conclusion, epigallocatechin‐3‐O‐gallate (EGCG) attenuated the endoglin expression and myocardial fibrosis by anti‐inflammatory effect *in vitro* and *in vivo*, the novel suppressive effect was mediated through JNK/AP‐1 pathway.

## Introduction

Myocardial fibrosis is a progressive pathological process accompanied by the abnormal thickening of the cardiac muscles, and the cardiac ventricular remodelling might result in myocardial fibrosis following injury 1. The results from past studies on the various mechanisms of myocardial injury, including myocardial infarction and hypertensive heart diseases, have reported myocardial fibrosis induction 2, 3. The decline in heart failure stimulates the compensatory pathways, and the sympathetic nervous system and the renin–angiotensin–aldosterone system are crucial and stimulate cardiomyocyte hypertrophy and myocardial fibrosis 4. Endoglin contains a larger extracellular (561 amino acids) and shorter cytosolic domains 5, and it is a homodimeric membrane glycoprotein co‐receptor of transforming growth factor‐β1 (TGF‐β1). Angiotensin II (Ang II) has been found to induce the endoglin expression in cultured cardiac fibroblasts (CFs), and also found in mice 6, 7 and patient 8 with heart failure.

Epigallocatechin‐3‐O‐gallate (EGCG) is documented to against several diseases or syndromes, including the anti‐cancer, anti‐hyperlipidemia, anti‐inflammation and anti‐hypertension effects 9. In previous study of developed hepatic fibrosis, EGCG counteracted the activity of TGF/SMAD, PI3 K/Akt/FoxO1 and NF‐κB pathways 10. Sheng *et al*. recently reported that the EGCG inhibited CFs proliferation was by increasing nitrite levels in cardiac hypertrophy rats 11. In addition, EGCG stimulated nitric oxide production *via* PI 3‐kinase‐dependent pathways, and it simultaneously improved cardiovascular pathophysiology in spontaneously hypertensive rats 12. Therefore, EGCG can be regarded as the components that contribute to the prevention of cardiovascular diseases by inflammation modulation; however, the precise molecular mechanism of EGCG on heart failure remains unclear. Therefore, the aim of this study was to elucidate the effect of EGCG on the myocardial fibrosis model and cultured Ang II‐induced CFs; in addition, the study investigated the myocardial fibrosis and endoglin molecular modulation. We demonstrated that EGCG attenuated myocardial fibrosis and heart failure by the suppressing endoglin and the JNK/AP‐1 pathway, and the anti‐inflammatory results suggested that it had the strong implications in controlling human cardiovascular diseases.

## Materials and methods

### Chemical reagents

The epigallocatechin‐3‐O‐gallate (EGCG) solution was prepared as a 10‐mmole/l stock in 100% dimethyl sulfoxide (DMSO). Matrigel basement membrane matrix was acquired from Becton Dickinson (Bedford, MA, USA). PD98059 (a specific and potent inhibitor of ERKs), SB203580 (a highly specific cell‐permeable inhibitor of p38 kinase) and SP600125 (a potent, cell‐permeable, selective and reversible c‐Jun N‐terminal kinase (JNK) inhibitor) were purchased from CALBIOCHEM^®^ (San Diego, CA, USA).

### Cell cultures

Cardiac fibroblasts (CFs) were prepared from the heart of adult male Wistar rats weighing 250–300 g using a method described previously 13. In brief, following the rapid excision of the hearts, the ventricles were isolated, minced, pooled and maintained in a solution of 100 U/ml collagenase I and 0.1% trypsin. The purity of these cultured CFs was >95%, as determined by positive staining for vimentin and negative staining for smooth muscle α‐actin, the von Willebrand factor, and desmin. All animals received humane care according to the guidelines stated in the *Guide for the Care and Use of Laboratory Animals*.

### Western blot analysis

Western blotting was performed as previously described 14. Rat anti‐endoglin monoclonal antibody and rabbit polyclonal anti‐JNK antibodies (Santa Cruz Biotechnology, Inc., CA, USA) were used. The signals were visualized by chemiluminescent detection. All Western blot analyses were quantified using densitometry.

### Real‐time reverse transcription‐polymerase chain reaction

Total RNA from the cultured fibroblasts was extracted using the single‐step acid guanidinium thiocyanate/phenol/chloroform extraction method. Real‐time RT‐PCR was performed as described previously 14. The rat endoglin primers were 5′‐ACCACTTCGGAAAAAGG‐3′ and 5′‐GCTGAAACGTGGGTCG ‐3′.

### RNA interference

The cultured 10^6^ CFs were transfected with 800 ng of endoglin siRNA oligonucleotides (Thermo Scientific, Waltham, MA, USA). Endoglin siRNA is a target‐specific 20–25 nt siRNA designed to knockdown endoglin gene expression, endoglin siRNA, and scrambled siRNA sequences GCUGGGAACCAUCGGCCAU and GCUGGGAAGGUAGGGCCAUUU.

### Electrophoretic mobility shift assay

An electrophoretic mobility shift assay (EMSA) was performed as described previously 15. Oligonucleotide sequences included the AP‐1 (activating factor 1) consensus binding region (5′‐CGCTTGATGACTCAGCCGGAA‐3′) and the AP‐1 mutant oligonucleotide sequence (5′‐CGCTTGATGACTTGGCCGGAA‐3′).

### Promoter activity assay

A −960 to +39 bp rat endoglin promoter construct was generated as follows. Rat genomic DNA was amplified with the forward primer 5′‐CCTGTCCCTCTTTGGAGACAG‐3′ and 5′‐CAGCAGGGTAATGACCAGAGG‐3′. The amplified product was digested with MluI and BglII restriction enzymes and ligated into a pGL3‐basic luciferase plasmid vector (Mission Biotech, Taipei, Taiwan) digested with the same enzymes. The endoglin promoter contains AP‐1 conserved sites (CCAGAC) at −795 to −794 bp. For the mutant, the AP‐1‐binding sites were mutated using the mutagenesis kit (Stratagene, La Jolla, CA, USA). Site‐specific mutations were confirmed by DNA sequencing. Plasmids were transfected into fibroblasts using a low pressure‐accelerated gene gun (Bioware Technologies, Taipei, Taiwan) essentially following the manufacturer protocol. Following 1 hr of Ang II stimulation, cell extracts were prepared using the Dual‐Luciferase Reporter Assay System (Promega) and measured for dual‐luciferase activity using a luminometer (Glomax Multi Detection System, Promega, Medison, WI, USA).

### Protein synthesis assay

Cardiac fibroblasts (CFs) were cultured in serum‐free medium in ViewPlate for 60 min. (Packard Instrument Co., Meriden, CT, USA). Ang II (10 ng/ml) was added to the medium. The cells were subsequently labelled with 100 μCi/ml ^3^H‐proline for 1 hr. EGCG or endoglin siRNA was added to the medium 30 min. before adding Ang II. The cells were washed twice with PB. MicroScint‐20 50 μl was added and the plate was read using TopCount (Packard Instrument Co.).

### Proliferation assay

Cardiac fibroblasts (CFs) were pre‐incubated with Ang II for 4 hrs before the proliferation assay. The ^3^H‐proline incorporation assay was performed as described previously 15. In brief, 5 × 10^3^ CF cells per well in serum‐free medium and incubated overnight. EGCG, SP600125, JNK1 siRNA and DMSO were subsequently added to the plate. Proline uptake was measured by adding 500 nCi/ml [3H]‐proline (Perkin Elmer, Boston, MA, USA). MicroScint‐20 (50 μl) was added, and the plate was read using TopCount (Packard Instrument).

### Confocal microscopy

The representative confocal microscopy was performed as described previously 15. In brief, the CF cells were fixed with 4% paraformaldehyde for 10 min. at room temperature (RT), permeabilized in permeabilization buffer for 5 min. and blocked in DAKO diluent for 30 min. at RT, to identify the localization of endoglin in the CFs cellular cytoplasm. Confocal microscopy at 400× magnification was performed with a Nikon confocal laser scanning microscope. Each experiment was conducted in triplicate.

### The acute myocardial infarction rat heart failure model and hemodynamic monitor

The proximal left anterior descending artery was ligated in adult Wistar rats to induce acute myocardial infarction (AMI). AMI and myocardial fibrosis were induced as previously described 16, 17. Sham‐operated control animals were prepared in similar manners, except that the aorta was left unpunctured. EGCG at 50 mg/kg 10, 15, 18 was administered one time daily using oral gavages for 14 days after AMI induction. The endoglin siRNA was administrated into left ventricular myocardium by a low pressure‐accelerated gene gun (Bioware Technologies, Taipei, Taiwan) while open for the rats' chest surgery, and followed the protocol from manufacturer essentially 7. In brief, 1600 ng was suspended in 4 μl of phosphate buffered saline (PBS) and then the siRNA‐containing solution was added to the loading hole near the nozzle. Pushing the trigger of the low pressure gene gun released the siRNA‐containing solution, which was directly propelled by helium at a pressure of 15 psi into the left ventricular myocardium of the rat. After 14 days since AMI induction, the rats were killed with an overdose of isoflurane. The left ventricular tissue was retrieved for Western blot analysis and immunohistochemical staining. Masson's trichrome staining was performed to delineate fibrosis tissue from the viable myocardium. All study protocols were approved by the Committee of Animal Care and Use of Shin Kong Wu Ho‐Su Memorial Hospital (permit number: 091217021) and were carried out according to the *Guide for the Care and Use of Laboratory Animals* (NIH publication No. 86‐23, revised 2011). The hemodynamic monitoring of rats was performed with polyethylene catheters for measurement through a Grass model tachograph preamplifier as previously described 19. The cardiac function of the rats was evaluated non‐invasively through an echocardiography performed with an Acuson Sequoia 512 ultrasound system (Siemens Medical Solutions, Malvern, PA, USA) with a 15‐MHz probe on the day the rats were killed, as well as 7 and 14 days after the surgery as previously described 13. The sonographer was blinded for the randomization of the rats. In this *in vivo* study, six rats were tested in each experiment group.

### Statistical analysis

The data were expressed as the mean ± SD. Statistical significance was determined by performing analysis of variance (anova) (GraphPad Software Inc., San Diego, CA, USA). The Dunnett's test and Mann–Whitney *U*‐test were conducted to compare multiple groups with a single control group. The Tukey–Kramer comparison test was performed for pairwise comparisons between multiple groups following anova. A *P* < 0.05 was considered statistically significant.

## Results

### Inhibitory effect of EGCG on Ang II‐induced endoglin expression in CFs

**Figure 1:**
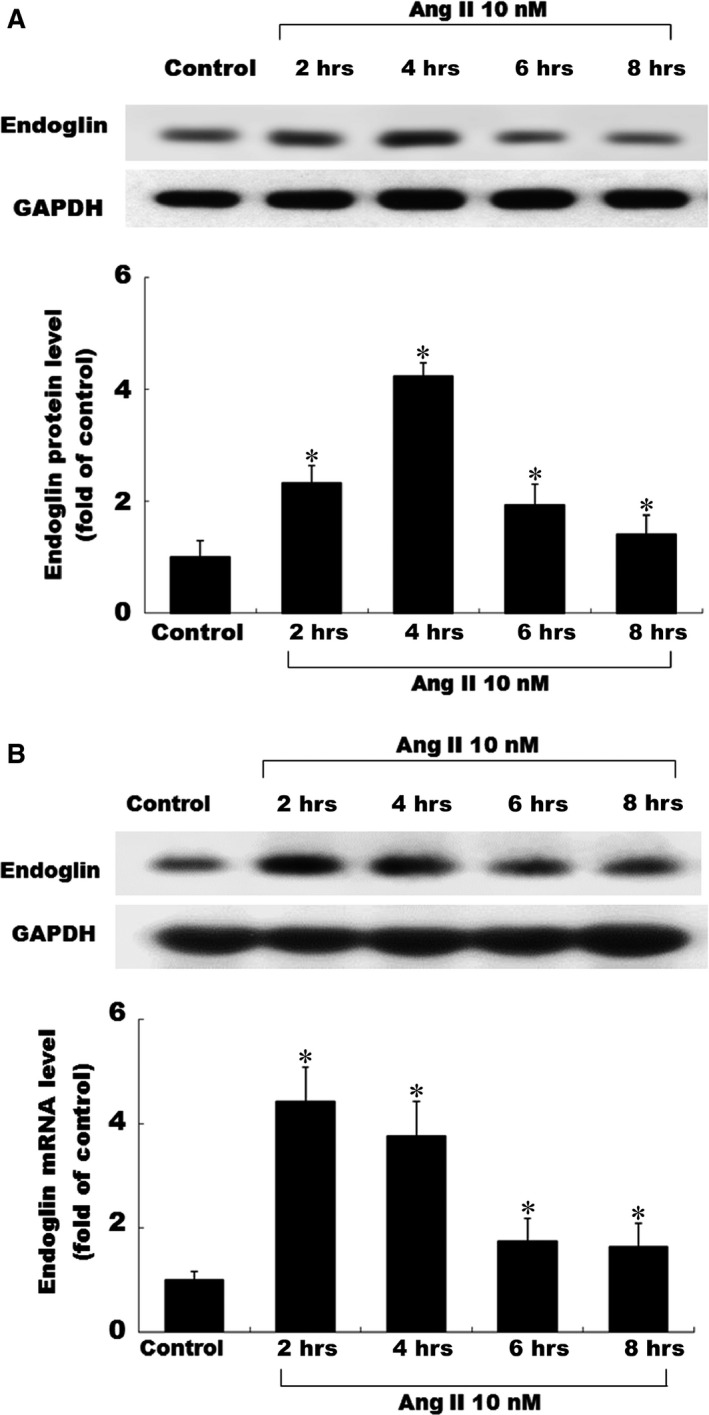
Effects of Ang II on the endoglin expression in cultured CFs. (**A**) Exogenous administration of angiotensin II (Ang II) increased the endoglin protein expression. Representative Western blot for endoglin in CFs following exogenous Ang II (10 nM) administration was for various periods of time. (**B**) Exogenous Ang II administration increased the endoglin mRNA expression in 4 hrs, and Epigallocatechin‐3‐O‐gallate attenuated endoglin expression in a concentration‐dependent manner. The detailed procedures for Western blot analysis and Northern blot analysis were described in Methods section. *P < 0.05 *versus* control (the repeated number was 3 per group). CFs, Cardiac fibroblasts.

**Figure 2:**
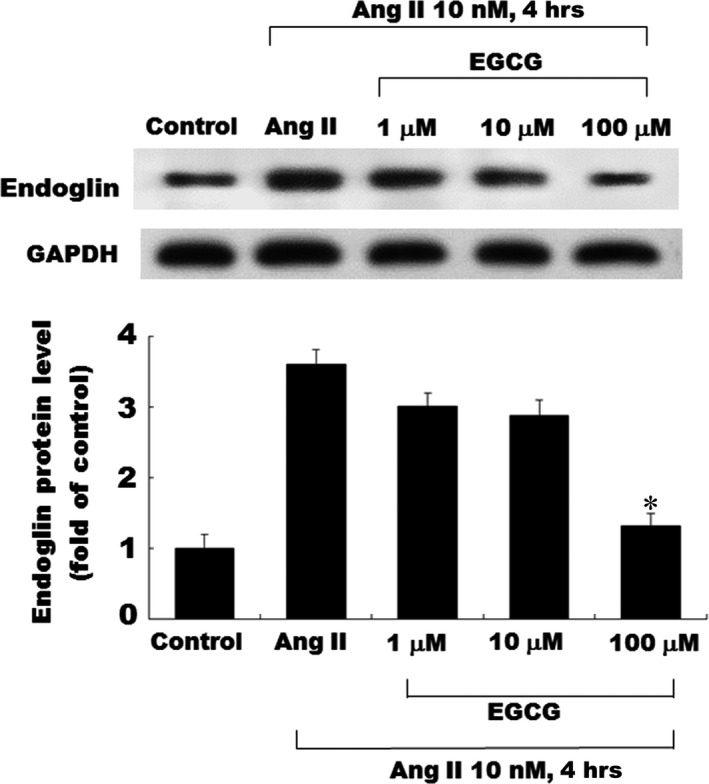
Inhibitory effects of EGCG on Ang II‐induced endoglin in cultured CFs. Representative Western blots for endoglin treated with various concentrations of EGCG in CFs following exogenous Ang II (10 nM) administration. EGCG attenuated the endoglin expression in a concentration‐dependent manner. *P < 0.05 *versus* control (the repeated number was 3 per group). CFs, Cardiac fibroblasts; EGCG, epigallocatechin‐3‐O‐gallate.

Cardiac fibroblasts (CFs) consistently expressed small amounts of endoglin; both mRNA and protein were detected in cultured CFs. Ang II had a significant stimulatory effect on both endoglin transcription and translation. The incubation of CFs with Ang II induced the expression of the endoglin protein in time course manners. The effect of Ang II on endoglin expression was also time dependent, as revealed by Northern blot analysis in Figure 1A. This effect of Ang II (10 nM) on endoglin protein expression peaked at 4 hrs. Figure 1B shows that EGCG inhibited the Ang II‐induced endoglin expression in a concentration‐dependent manner. EGCG exerted an inhibitory effect of Ang II‐induced endoglin expression in concentration‐dependent manners (Fig. 2).

### Molecular signalling regulation of Ang II‐induced endoglin expression on EGCG in CFs

**Figure 3:**
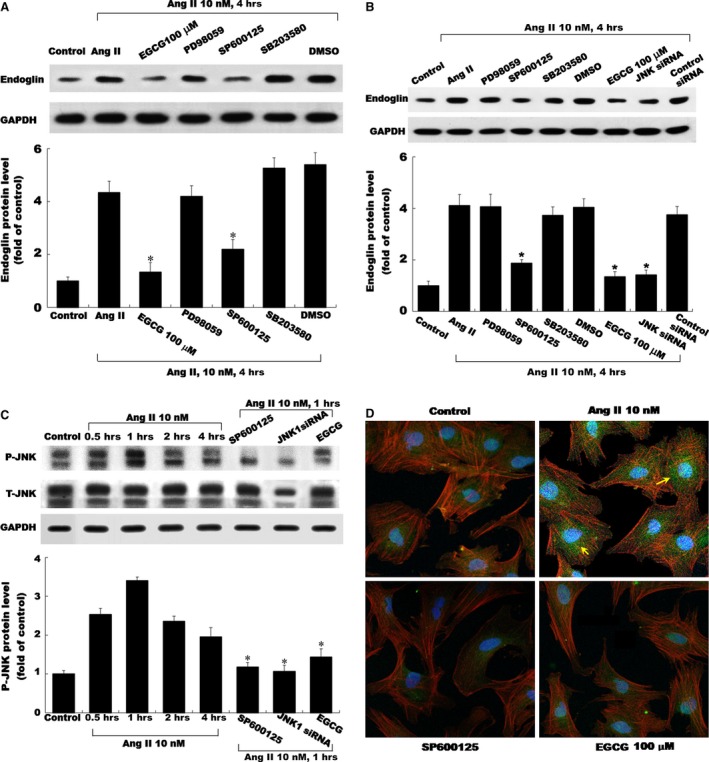
Molecular signalling regulation of Ang II‐induced endoglin in CFs. Representative Western blots for the endoglin protein levels in CFs subjected to Ang II induction for 4 hrs or control cells without Ang II induction in both the absence and presence of inhibitors, EGCG and siRNA. EGCG (100 nM) and inhibitor SP600125 revealed inhibitory effects on the endoglin expression in a concentration‐dependent manner (**A**,** B** and **C**). (**D**) Endoglin of serum‐starved CFs was stained with tetramethylrhodamine isothiocyanate–phalloidin (green dot) and observed through inverse fluorescence microscopy. Representative imaging showing endoglin protein levels (green dot) were apparently decreased and changed with either JNK inhibitor (SP600125) or EGCG (100 nM) treatment compared with the Ang II‐stimulated plate. Original magnification, ×200. The values from stimulated CFs have been normalized to those in the control group. *P < 0.05 *versus* control (the repeated number was 3 per group). CFs, cardiac fibroblasts; EGCG, epigallocatechin‐3‐O‐gallate.

The regulatory effect of EGCG on Ang II‐induced endoglin expression in CFs was evaluated; addition of EGCG (100 μM), or SP600125, significantly attenuated the endoglin protein expression induced by Ang II (10 nM) (Fig. 3A, B and C). PD98059 and SB203580 did not affect endoglin protein expression induced by Ang II. Finally, confocal microscopy revealed the endoglin expression in CFs following EGCG treatment (Fig. 3D). Endoglin protein (green) level increased in the cytoplasm of CFs following Ang II induction for 4 hrs. EGCG and SP600125 suppressed the expression of endoglin induced by Ang II.

### Ang II increased AP‐1‐binding activity and EGCG inhibited promoter activity

**Figure 4:**
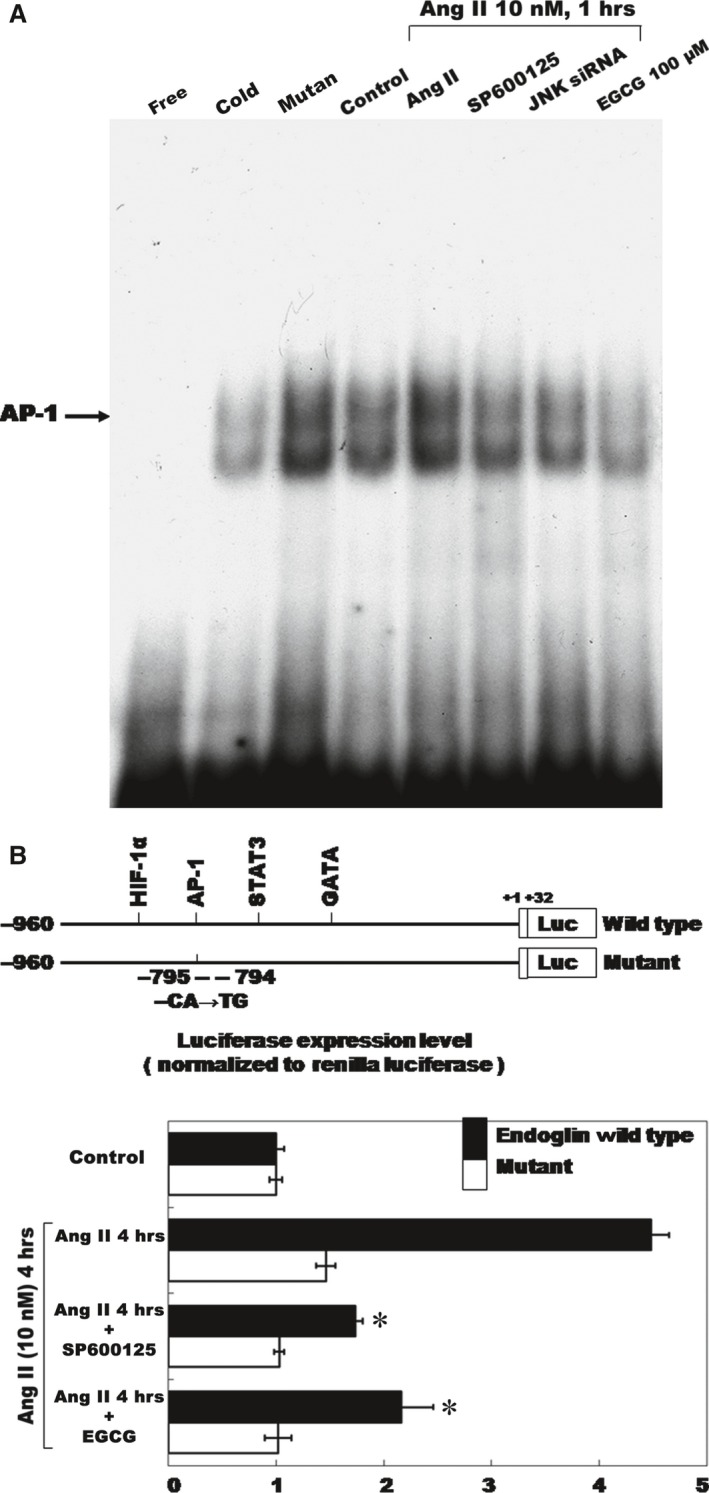
Angiotensin II‐endoglin expression increased the AP‐1‐binding activity and EGCG inhibits promoter activity. (**A**) Representative electrophoretic mobility shift assay showing protein binding to the AP‐1 oligonucleotide in the nuclear extracts of CFs following Ang II induction both in the presence and in the absence of either EGCG (100 μM) or JNK1 inhibitor (SP600125). Similar results were observed in two other independent experiments. A significant supershifted complex (super) following the incubation with AP‐1 antibody was observed. Cold oligo means unlabelled AP‐1 oligonucleotides. (**B**) Constructs of AP‐1 promoter gene. Positive +1 demonstrates the initiation site for the endoglin transcription. Mutant endoglin promoter indicates mutation of AP‐1‐binding sites in the endoglin promoter as indicated. Quantitative analysis of the resistin promoter activity. Cultured CFs were transiently transfected with the pAP‐1‐Luc by gene gun. The luciferase activity in cell lysates was measured and normalized with the renilla activity (*n* = 3 per group). *P < 0.05 *versus* control. CFs, cardiac fibroblasts; EGCG, epigallocatechin‐3‐O‐gallate.

The EMSA assay revealed that Ang II increased AP‐1 DNA‐protein‐binding activity (Fig. 4A). An excess of unlabelled AP‐1 oligonucleotide competed with the probe for binding to AP‐1 proteins, whereas an oligonucleotide containing a 2‐bp substitution in the AP‐1‐binding site did not compete for binding. The addition of SP600125 and EGCG 30 min. before Ang II (10 nM) induction diminished the DNA‐protein‐binding activity induced by Ang II. DNA‐binding complexes induced by Ang II could be supershifted by a monoclonal AP‐1 antibody, indicating the presence of this protein in these complexes. We cloned the promoter region of rat endoglin (−795 to +22), and constructed a luciferase reporter plasmid (pGL3‐Luc), to examine whether endoglin expression induced by Ang II is regulated at the transcription level. As shown in Figure 4B, transient transfection experiment in CFs with this reporter gene revealed that Ang II induction for 4 hrs significantly caused substantial endoglin promoter activation. This result revealed that endoglin expression was induced at transcription level by Ang II. When the AP‐1‐binding sites were mutated, the increased promoter activity induced by Ang II was abolished. Moreover, the addition of SP600125 and EGCG caused an inhibition in endoglin transcription. These results suggested that the AP‐1‐binding site in the endoglin promoter is essential for transcriptional regulation by Ang II and that Ang II regulated the endoglin promoter *via* JNK pathways.

### Attenuated proliferation effects of EGCG, SP600125, and JNK1 siRNA on CFs

**Figure 5:**
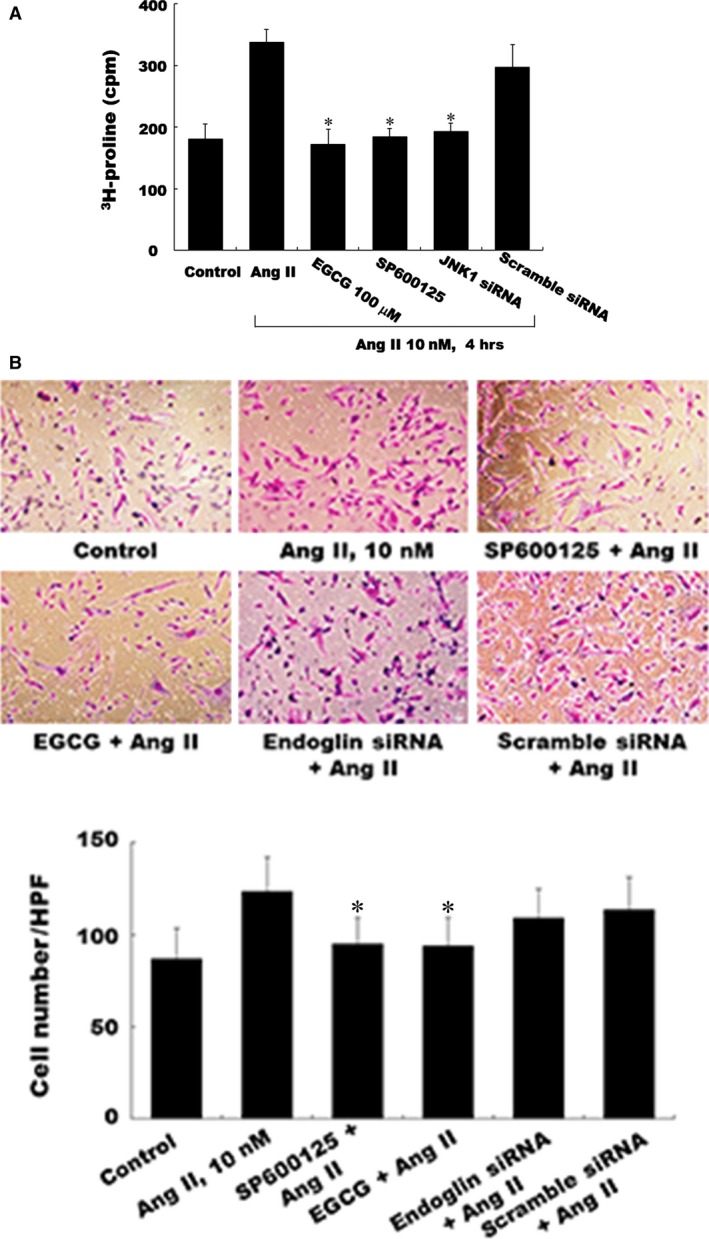
Effects of EGCG, JNK inhibitor, and a JNK siRNA on the proliferation assay in Ang II‐induced CFs. (**A**) Representative [3H]‐proline incorporation assay showing the proliferation of CFs following Ang II (10 nM) stimulation both in the presence and in the absence of either EGCG (100 μM) or JNK inhibitor SP600125 or JNK1 siRNA. (**B**) EGCG or endoglin siRNA attenuates the migration activity. The assay was performed in triplicate, and the values from the induced CFs have been normalized to those from the control cells (*n* = 4 per group). *P < 0.05 *versus* control. CFs, cardiac fibroblasts; EGCG, epigallocatechin‐3‐O‐gallate.

The [3H]‐proline incorporation assay was performed to investigate the effects of EGCG on the Ang II‐induced CF proliferation. Figure 5A shows that treatment with Ang II increased the CF proliferation significantly. Pre‐treatment with EGCG, SP600125, or JNK1 siRNA significantly attenuated the CF proliferation, which was stimulated by Ang II. Moreover, the similar result was obtained from the migration assay (Fig. 5B).

### EGCG and endoglin siRNA attenuated the AMI‐related endoglin expression *in vivo*

**Figure 6:**
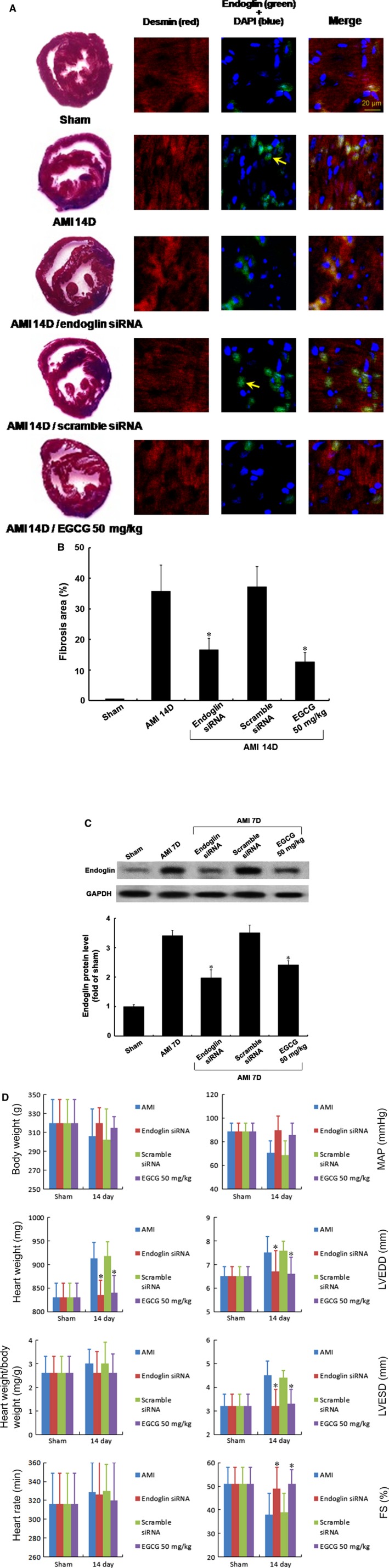
Immunohistochemnical staining of the left ventricular myocardium after induction of acute myocardial infarction (AMI) and hemodynamic monitor by treatment with/without EGCG or endoglin siRNA. Significantly increased immunoreactive signals were observed for endoglin following AMI for 14 days. (**A**) Left panel, representative cross‐section of the cardiac ventricle stained with the haematoxylin by EGCG (50 mg/kg) treatment or endoglin siRNA. The right three panels showed double staining for endoglin siRNA (green colour) by fibroblasts actin labelling (red colour). (**B**) Quantitative analysis of the cross‐sectional area of the endoglin protein expression after AMI for 14 days. (**C**) Quantitative analysis of the cross‐sectional myocardial fibrosis size measured, EGCG and endoglin siRNA significantly decreased the myocardial fibrosis induced following AMI for 7 days. Rare endoglin signals were observed in the sham group. (**D**) EGCG and endoglin siRNA attenuated the heart weight, the left ventricular end‐diastolic dimension (LVEDD), the left ventricular systolic–diastolic dimension (LVESD) and the fraction shortening (FS). *P* < 0.05 *versus* control. EGCG, epigallocatechin‐3‐O‐gallate.

The AMI‐related myocardial fibrosis model used to mimic heart failure for 14 days, increased the fibrotic area and ventricular volume significantly, compared with those in the control group (Fig. 6A, left panel). Endoglin siRNA delivery or EGCG treatment attenuated the fibrotic area significantly following AMI. The right three panels in Figure 6A show that the immunoreactive signals for endoglin and with EGCG treatment attenuated endoglin presentation following AMI for 14 days. The fibrotic area reduced by 53.4% by endoglin siRNA delivery and reduced by 64.5% by EGCG treatment (Fig. 6B). Control siRNA did not reduce the fibrotic area following AMI injury. Similarly, endoglin siRNA delivery or EGCG treatment significantly decreased the endoglin protein presentation following AMI injury for 7 days (Fig. 6C).

### Endoglin siRNA and EGCG attenuated AMI‐related heart failure

Rat hemodynamic monitoring was performed with polyethylene catheters for measurement using a Grass model tachograph preamplifier as described previously 18, and the data indicated that the heart weight, ratio of heart weight/bodyweight, mean arterial pressure (MAP), left ventricular end‐diastolic dimension (LVEDD), left ventricular end‐systolic dimension (LVESD) and friction shortening (FS) increased significantly following AMI for 14 days. Treatment with either endoglin siRNA or EGCG attenuated the heart weight, heart weight/bodyweight ratio, LVEDD, LVESD and FS (Fig. 6D).

## Discussion

**Figure 7:**
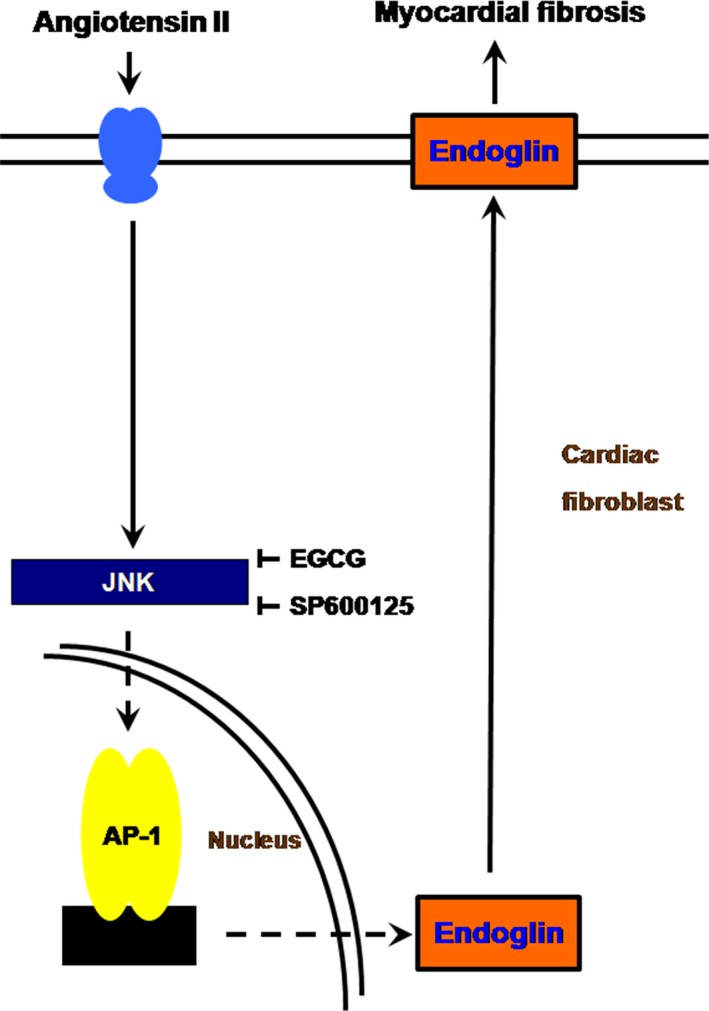
Suppressive molecular mechanism of EGCG on myocardial fibrosis *in vitro* and *in vivo*. The graphic content presented here indicated that EGCG suppresses AngII‐induced endoglin through modulation of JNK/AP‐1 pathway in cultured cardiac fibroblasts and acute myocardial infarction‐related myocardial fibrosis. This study revealed the evidence of EGCG attenuated the endoglin expression and myocardial fibrosis by anti‐inflammatory effect *in vitro* and *in vivo*. EGCG, epigallocatechin‐3‐O‐gallate.

Green tea has been reported to promote health for thousand years in Asia. Accumulating scientific reports have recently documented the potential benefits of green tea, and it contains the abundance of catechin and EGCG 20. Increasing evidence has shown that EGCG has protective effects against cardiovascular and metabolic syndromes. Several lines of evidence have pointed out that EGCG regulates anti‐inflammatory pathways in both *in vivo* and *in vitro* models 21, 22. This study investigated the effect of EGCG on ventricular myocardial fibrosis and its molecular regulating mechanisms for the first time. Treated MI‐induced heart failure (HF) rats with either tyrosine phosphorylation‐regulated kinase 1A (Dyrk1A) inhibitor or EGCG improved the HF symptoms and reversed the molecular changes of Dyrk1A 23. Moreover, EGCG reduced the presentation of IL‐6 and TNF‐α in the ischaemia/reperfusion injury model by inhibiting NF‐κB 24. Our data revealed that EGCG inhibited the ventricular fibrosis through endoglin decrease and JNK inhibition. Results from full spectrum studies have revealed that EGCG enriched the potential of anti‐inflammation modulation in cardiovascular disorders (Fig. 7).

A previous study indicated that endoglin was up‐regulated through Ang II type 1 (AT_1_) and MAPKp42/44 in Ang II‐induced cultured CFs and further indicated that endoglin was crucial in Ang II‐induced cardiac remodelling 6. Li *et al*. demonstrated that Ang II increased the expression of endoglin mRNA and protein, with no effect on TGF‐β receptors I and II expression in CF cultures 6. Meanwhile, they also revealed that Ang II induced endoglin and also through AT1 activation increased the expression of TGF‐β1 type 1 and type 2 receptors in Ang II‐induced endothelial cell cultures 25. Another previous study reported that plasma endoglin was a marker for predicting cardiovascular events in patients with coronary artery disease following percutaneous coronary intervention 26. Therefore, targeted endoglin therapy for preventing myocardial fibrosis and heart failure may improve the clinical outcome in patients with heart failure. Loss of the PI3K/Akt and/or HO‐1 activity promoted soluble endoglin release and led to endothelial dysfunction in endothelial cell cultures 27. In a mouse model of right ventricular (RV) pressure overload, endoglin was present as a regulator of TGF‐β1 signalling by calcineurin and canonical transient receptor protein channel 6 (TRPC‐6) in the RV, and it was present as a potential therapeutic target limiting RV fibrosis 28. Endoglin down‐regulation of TGF‐β1‐induced collagen synthesis was dependent on ERK1/2 MAPK activation in L6E9 myoblast cultures 29. In our study, EGCG treated the Ang II‐induced cardiac fibroblast model attenuated endoglin expression through inhibiting phosphorated JNK and AP‐1 activity*,* but did not abolished ERK or p38 MAPK. Moreover, the similar result was also revealed in AMI‐treated rats.

Compared with the EGCG‐treated cardiac hypertrophy model 11, our rat left coronary artery ligation model was designed to mimic the pathophysiological condition of AMI‐related myocardial fibrosis and heart failure. The myocardial fibrosis model in our study augmented the left ventricular wall mass, and ratio of heart weight over bodyweight, but not increased the bodyweight or heart rate. According to the study of cardiac function monitor by left ventricular assist device implantation, the degree of cardiac fibrosis and myocyte size had significant correlations with changes in LVEDD and LVESD 30. Our study provided further hemodynamic or structure data to investigate the effect of EGCG. Furthermore, accumulating evidence has indicated the effect of herbal products on myocardial fibrosis and heart failure, and that oxymatrine (extracted from a traditional Chinese herb *Sophora japonica*) protected against myocardial fibrosis through the modulation of the TGF‐β(1)‐Smads signal pathway following AMI for 4 weeks 31. In this study, our data indicated that EGCG and endoglin siRNA reduced JNK/AP‐1 expression in rats 14 days after AMI. In addition, the endoglin protein alleviated significantly in rats 7 days after AMI (Fig. 6C), and it was attenuated by EGCG, but was revealed as discordant with hemodynamic changes, the inhibitory effect on endoglin protein expression present earlier than the pathophysiologic alteration. In this study, our data suggest that EGCG could potentially promote cardiovascular health through various anti‐inflammatory mechanisms.

In conclusion, we showed that EGCG attenuated endoglin expression and myocardial fibrosis *in vitro* and *in vivo*, and that the novel suppressive effect was mediated through endoglin/JNK/AP‐1 pathway, which suggested the strong implications in controlling human cardiovascular diseases.

## Conflict of interest

The authors confirm that there are no conflicts of interest.
